# 

**Published:** 1918-12-21

**Authors:** 


### THE COLLEGE OF NURSING

### (Limited by Guarantee).

EDINBURGH CENTRE.

ADDRESS BY MRS. ALTENTOP.

The Edinburgh Centre of the College of Nursing was fortunate in having the Organising Representative of the National Council for Combating Venereal Diseases to address its meeting on Tuesday, December 10. Miss Gordon, Matron of the Hospice, introduced Mrs. Altentop, whose subject was "Social Prevention." Besides urging the necessity for the education of all classes of society regarding venereal diseases, Mrs. Altentop sketched some of the social reforms which would go far to lessen the evil in our midst. These include hostels where women, whether married or not, could go for their confinement and for treatment during pregnancy, and hostels where young boys and girls engaged in industrial employment could live under decent conditions. The housing question was, she said, of vital importance in combating venereal diseases. At the present day venereal diseases were spread more by the "flapper" or "amateur" than by the generic prostitute. Hundreds of young girls gave their bodies out of ignorance—for a lark, or perhaps because of the glamour of khaki. Our young people must have healthy, wholesome recreation after their day's work; mixed clubs, where the members danced, and preferably made their own rules, did much for young men and girls, but they should be of a purely social and secular character, as were the entertainments provided for members of the upper classes. The work of women patrols could also do much to help to stamp out venereal diseases. We must all do our utmost to suppress them; and nurses could do a great deal, especially when they believe that the evil can be eradicated. Miss Brunton, Secretary of the Scottish Board of the College, proposed a very hearty vote of thanks to Mrs. Altentop.

### Passing Events and Topics.

### Royal Dental Hospital of London.

Lord Burnham has accepted the post of trustee of the Royal Dental Hospital, Leicester Square, in conjunction with Lord Kinnaird and Mr. F. A. Bevan, in place of the late Mr. Richard Winch.

### The Three Lord Mayors.

Three of the Lord Mayors of London, Sir Horace Marshall, Sir Charles Hanson, Bt., and Sir George Truscott, Bt., have severally written a message of appeal for the Royal Hospital and Home for Incurables, Putney Heath. These messages are being sent out in the hope of paying off a debt of £3,000.

### The Elsie Inglis Chair of Medicine.

The Committee of the Elsie Inglis London Memorial Fund hopes to hand over the money raised for the endowment of a Chair of Medicine in the University of Belgrade on the day of the return of the Serbian Government to its capital. The required sum of £12,000 is not yet complete, and the Hon. Treasurers, the Countess of Selborne and Miss Teresa Gosse, 66 Victoria Street, S.W. 1, appeal to donors to send their contributions quickly. The Elsie Inglis Chair of Medicine will be the first gift from this country towards the reconstitution of the national university.

### The Hospital Saturday Fund.

The Council of the Hospital Saturday Fund (London) has received a cheque for £1,000 from Gordon Campbell, Esq., chairman of the Collection Committee of the British Red Cross Society, in connection with the Entertainments Industry Section of the Lord Mayor's Appeal. It will be remembered that during October last the Hospital Saturday Fund refrained from collecting at places of entertainment, so as not to clash with the special collections then being made on behalf of the British Red Cross Society. The cheque now received for £1,000 has been sent so that the Hospital Saturday Fund should not suffer by reason of these special collections.

### Gifts and Bequests.

Miss Harrison, of Dean Hill, Matlock, has left £12,500 to charities, including £2,500 to the Church Missionary Society for a missionary in India.

Miss Maria Strachan, of Bayswater, has left £1,000 to the Scottish Hospital, Crane Court; £300 to the Royal Hospital for Incurables, Putney; £200 to the Paddington Green Children's Hospital, and the ultimate residue of her property to King Edward's Hospital Fund.

Mr. W. W. Brocklehurst, of Chester, left estate to the value of £197,849. Amongst the legacies were £1,000 each to the Macclesfield Infirmary and the Macclesfield Certified Midwives' School, and £500 to the Asylum for Deaf and Dumb Children, Old Kent Road.

A number of bequests were made by the late Mr. T. N. Alexander, of South Shields, including £1,000 each to St. Dunstan's and the Ingham Infirmary, South Shields, £300 to Dr. Barnardo's Homes, and £100 to King Edward's Hospital Fund for London.

Mr. G. H. Harrison, of Buckingham, left £100 to the Buckingham Nurses' Home.

As an armistice thankoffering Mr. W. T. Farr, of Mumbles, has given £5,000 to Swansea Hospital.

The Chelsea Hospital for Women has received a grant of £700 from the trustees of the Zunz Bequest, and a donation of £100 from J. F. Yarrow, Esq., in response to the Costless Peace Appeal.

Mr. J. B. Dugdale, whose estate was valued at £202,932, left £5,000 to King Edward's Hospital Fund and £1,000 each to the Dorset County Hospital and the Victoria Hospital, Bournemouth.

Major-General W. H. Knight, late 48th Regiment, of Dover, has left £500 to the Royal Victoria Hospital, Dover, and the residue of his property for such institutions for the benefit of soldiers (not commissioned officers) who are fighting in the war as the executors may determine.

### Matrons' and Sisters' Appointments.

ST. MARY ISLINGTON INFIRMARY, HIGHGATE HILL.—Miss Elsie Worvell has been appointed sister. She was trained at the above-named institution, holds the certificate of the Central Midwives Board, and has been staff nurse at Queen Charlotte's Hospital, Marylebone Road.

BOLTON INFIRMARY.—Miss Edith Worsley Lyon has been appointed out-patient sister. She was trained at the above-mentioned institution and at the Hospital for Infectious Diseases, Fazakerley, Liverpool. On the completion of her training she was appointed sister of a military ward at Bolton Infirmary, and later became night superintendent at the City Hospital North, Liverpool.

COTTAGE HOSPITAL, PETWORTH.—Miss E. J. Whiteside has been appointed nurse-matron. She was trained at Crumpsall Workhouse Infirmary, Manchester, has been charge sister at the Park Fever Hospital, Hither Green, and matron of the Cottage Hospital, Bourton-on-the-Water. She has also done Army nursing in France.

